# *threo*-2-(2,6-Dimethoxy­phen­oxy)-1-(4-eth­oxy-3-methoxy­phen­yl)propane-1,3-diol

**DOI:** 10.1107/S1600536809018431

**Published:** 2009-05-23

**Authors:** Kentaro Ishizuka, Daisuke Ando, Takashi Watanabe, Masaharu Nakamura

**Affiliations:** a International Research Center for Elements Science, Institute for Chemical Research, Kyoto University, Uji, Kyoto 611-0011, Japan; b Institute of Sustainability Science, Kyoto University, Japan; c Research Institute for Sustainable Humanosphere, Kyoto University, Uji, Kyoto 611-0011, Japan

## Abstract

In the crystal structure of the title compound, C_20_H_26_O_7_, a lignin model compound, the asymmetric unit contains two mol­ecules which adopt almost identical overall conformations with some deviation in the region of the terminal hydroxyl groups. The two mol­ecules are linked by an inter­molecular O—H⋯O hydrogen bond. They also develop intra­molecular O—H⋯O hydrogen bonds.

## Related literature

For the synthesis, see: von Unge *et al.* (1988 ▶). For the crystal structure of two related guaiacyl­glycerol *β*-syringyl ether type lignin model dimers, see: Langer, Li *et al.* (2002 ▶); Langer, Lundquist *et al.* (2002 ▶). For the crystal structure of several related syringylglycerol *β*-syringyl ethers, see: Langer & Lundquist (2001 ▶); Langer, Li *et al.* (2002 ▶); Langer, Lundquist *et al.* (2002 ▶); Langer *et al.* (2005 ▶); Lundquist *et al.* (2005 ▶); Stomberg & Lundquist (1989 ▶).
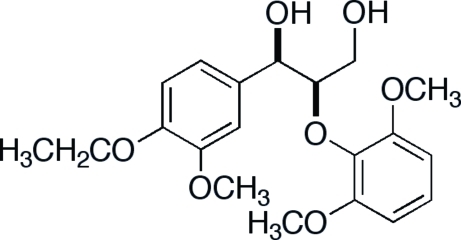


## Experimental

### 

#### Crystal data

C_20_H_26_O_7_
*M* *_r_* = 378.42
Monoclinic, 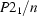

*a* = 9.929 (4) Å
*b* = 28.066 (10) Å
*c* = 13.985 (5) Å
β = 100.954 (2)°
*V* = 3826 (2) Å^3^
*Z* = 8
Mo *K*α radiation
μ = 0.10 mm^−1^
*T* = 173 K
0.50 × 0.50 × 0.40 mm

#### Data collection

Rigaku Saturn diffractometer
Absorption correction: numerical (**CrystalClear**; Rigaku, 2006 ▶) *T*_min_ = 0.938, *T*_max_ = 0.961
30389 measured reflections
8726 independent reflections
3941 reflections with *F* ^2^ > 2σ(*F* ^2^)
*R*_int_ = 0.056

#### Refinement

*R*[*F* ^2^ > 2σ(*F* ^2^)] = 0.074
*wR*(*F* ^2^) = 0.227
*S* = 1.00
8726 reflections
539 parameters
H-atom parameters constrained
Δρ_max_ = 0.59 e Å^−3^
Δρ_min_ = −0.67 e Å^−3^

### 

Data collection: *CrystalClear* (Rigaku, 2006 ▶); cell refinement: *CrystalClear*; data reduction: *CrystalStructure* (Rigaku/MSC, 2007 ▶); program(s) used to solve structure: *SIR2004* (Burla *et al.*, 2005 ▶); program(s) used to refine structure: *CRYSTALS* (Carruthers *et al.*, 1999 ▶); molecular graphics: *ORTEP-3 for Windows* (Farrugia, 1997 ▶); software used to prepare material for publication: *CrystalStructure*.

## Supplementary Material

Crystal structure: contains datablocks global, I. DOI: 10.1107/S1600536809018431/bx2203sup1.cif

Structure factors: contains datablocks I. DOI: 10.1107/S1600536809018431/bx2203Isup2.hkl

Additional supplementary materials:  crystallographic information; 3D view; checkCIF report

## Figures and Tables

**Table 1:** Hydrogen-bond geometry (Å, °)

*D*—H⋯*A*	*D*—H	H⋯*A*	*D*⋯*A*	*D*—H⋯*A*
O3—H3⋯O6	0.82	2.09	2.772 (3)	141
O4—H4⋯O7	0.82	2.46	2.758 (3)	103
O10—H10⋯O13	0.82	2.17	2.878 (3)	145
O11—H11⋯O4	0.82	2.19	2.882 (3)	142
O3—H3⋯O5	0.82	2.44	2.859 (3)	113
O10—H10⋯O12	0.82	2.32	2.760 (3)	114
O11—H11⋯O12	0.82	2.36	2.795 (3)	114

**Fig. 1.**
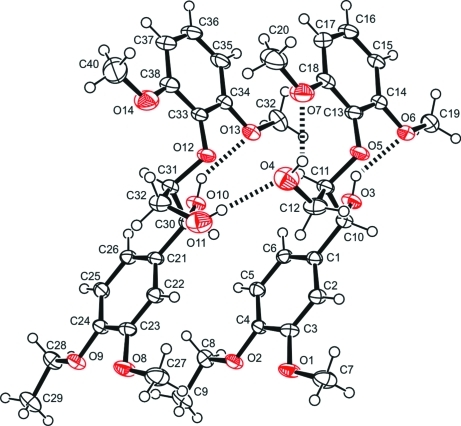
A view of the molecular structure of the title compound, which is an asymmetric unit with two molecules, with the atom-labeling scheme. Displacement ellipsoids are drawn at the 50% probability level. Dash lines indicate hydrogen bonds.

## supplementary crystallographic information

### Comment

Lignin is a complex biopolymer consisting of *p*-hydroxyphenyl, guaiacyl and syringyl phenylpropanoid monomer units. Aryl glycerol *β*-syringyl ethers are the main structural elements in hardwood lignins. The crystal structure of *threo*-2-(2,6-dimethoxyphenoxy)-1-(3,4-dimethoxyphenyl)-1,3- propanediol (Langer, Li *et al.*, 2002; Langer, Lundquist *et al.*, 2002) and several syringylglycerol *β*-syringyl ethers are reported. We have prepared a guaiacylglycerol *β*-syringyl ether type lignin model dimer having an ethyl ether substituent at 4-position, which does not show a similar packing structure or hydrogen bonding network to that of the methyl ether counterpart.

There are two molecules of the title compound in the asymmetric unit (Fig. 1). The conformations of two molecules are similar, as indicated by the torsion angles of 59.1 (3) and 61.3 (3)° along the phenylpropanoid skeleton (C1—C10—C11—C12 and C21—C30—C31—C32), respectively. They are connected with each other by the hydrogen bond of O11—H11···O4 through the terminal hydroxyl groups. Intramolecular hydrogen bonds of O3—H3···O6, O4—H4···O7 and O10—H10···O13 are also present (Table 1).

### Experimental

The title compound was synthesized in two steps with a procedure similar to that reported in the literature (von Unge *et al.*, 1988). Separation of the *erythro* and *threo* forms of the first step product was accomplished by recrystallization from methanol to give *threo*-3-(4-ethoxy-3-methoxyphenyl)-2-(2,6-Dimethoxyphenoxy)- -3-hydroxypropanoic acid 1. The title compound, *threo*-1-(4-ethoxy-3-methoxyphenyl)-2-(2,6-dimethoxyphenoxy)-1,3- propanediol, was synthesized from 1, and Single crystals of that suitable for X-ray crystallography were obtained from methanol.

### Refinement

All H atoms were positioned geometrically and treated as riding, with C—H bond lengths constrained to 0.93 (aromatic), 0.96 (methyl), 0.97 (methylene), 0.98 (methine) and 0.82 Å (hydroxyl), and with *U*_iso_(H) = *xU*_eq_ (carrier atom), where *x* = 1.5 for methyl and hydroxyl groups and *x* = 1.2 for other H atoms.

### Crystal data

**Table:**

C_20_H_26_O_7_	*F*(000) = 1616.00
*M**_r_* = 378.42	*D*_x_ = 1.314 Mg m^−^^3^
Monoclinic, *P*2_1_/*n*	Mo *K*α radiation, λ = 0.71070 Å
Hall symbol: -P 2yn	Cell parameters from 7455 reflections
*a* = 9.929 (4) Å	θ = 3.0–27.5°
*b* = 28.066 (10) Å	µ = 0.10 mm^−^^1^
*c* = 13.985 (5) Å	*T* = 173 K
β = 100.954 (2)°	Block, colorless
*V* = 3826 (2) Å^3^	0.50 × 0.50 × 0.40 mm
*Z* = 8	

### Data collection

**Table:**

Rigaku Saturn diffractometer	3941 reflections with *F*^2^ > 2σ(*F*^2^)
Detector resolution: 7.31 pixels mm^-1^	*R*_int_ = 0.056
ω scans	θ_max_ = 27.5°
Absorption correction: numerical (*CrystalClear*; Rigaku, 2006)	*h* = −11→12
*T*_min_ = 0.938, *T*_max_ = 0.961	*k* = −35→36
30389 measured reflections	*l* = −18→16
8726 independent reflections	

### Refinement

**Table:**

Refinement on *F*^2^	H-atom parameters constrained
*R*[*F*^2^ > 2σ(*F*^2^)] = 0.074	*w* = 1/[0.0018*F*_o_^2^ + σ(*F*_o_^2^)]/(4*F*_o_^2^)
*wR*(*F*^2^) = 0.227	(Δ/σ)_max_ < 0.001
*S* = 1.00	Δρ_max_ = 0.59 e Å^−^^3^
8726 reflections	Δρ_min_ = −0.67 e Å^−^^3^
539 parameters	

### Special details

**Table:**

Refinement. Refinement was performed using all reflections. The weighted *R*-factor (*wR*) and goodness of fit (*S*) are based on *F*^2^. *R*-factor (gt) are based on *F*. The threshold expression of *F*^2^ > 2.0 σ(*F*^2^) is used only for calculating *R*-factor (gt).

### Fractional atomic coordinates and isotropic or equivalent isotropic displacement parameters (Å^2^)

**Table:**

	*x*	*y*	*z*	*U*_iso_*/*U*_eq_	
O(1)	0.4275 (2)	0.14739 (7)	0.32648 (14)	0.0342 (6)	
O(2)	0.6049 (2)	0.07942 (7)	0.34454 (14)	0.0303 (5)	
O(3)	0.3267 (2)	0.07866 (7)	0.71410 (14)	0.0320 (6)	
O(4)	0.5503 (2)	0.23444 (8)	0.73756 (17)	0.0475 (7)	
O(5)	0.3404 (2)	0.16987 (8)	0.80643 (14)	0.0327 (6)	
O(6)	0.1968 (2)	0.09850 (8)	0.86837 (14)	0.0347 (6)	
O(7)	0.5527 (2)	0.21120 (9)	0.92951 (16)	0.0478 (7)	
O(8)	0.9339 (2)	0.15184 (8)	0.33101 (15)	0.0372 (6)	
O(9)	1.1062 (2)	0.08238 (7)	0.34465 (14)	0.0308 (5)	
O(10)	0.8242 (2)	0.07441 (7)	0.71045 (14)	0.0307 (5)	
O(11)	0.7854 (2)	0.22309 (8)	0.64640 (16)	0.0475 (7)	
O(12)	0.8095 (2)	0.16035 (7)	0.80390 (14)	0.0296 (5)	
O(13)	0.6895 (2)	0.08724 (8)	0.87353 (15)	0.0357 (6)	
O(14)	1.0163 (2)	0.20864 (9)	0.91436 (17)	0.0518 (8)	
C(1)	0.4133 (3)	0.10906 (10)	0.5791 (2)	0.0241 (7)	
C(2)	0.3838 (3)	0.13478 (10)	0.49118 (19)	0.0260 (7)	
C(3)	0.4498 (3)	0.12462 (10)	0.4152 (2)	0.0262 (7)	
C(4)	0.5457 (3)	0.08738 (10)	0.42410 (19)	0.0242 (7)	
C(5)	0.5741 (3)	0.06160 (10)	0.5105 (2)	0.0262 (7)	
C(6)	0.5080 (3)	0.07258 (10)	0.5872 (2)	0.0253 (7)	
C(7)	0.3190 (3)	0.18146 (11)	0.3079 (2)	0.0390 (9)	
C(8)	0.6995 (3)	0.04042 (10)	0.3506 (2)	0.0296 (8)	
C(9)	0.7502 (3)	0.03888 (13)	0.2561 (2)	0.0419 (10)	
C(10)	0.3426 (3)	0.12164 (10)	0.6632 (2)	0.0271 (7)	
C(11)	0.4245 (3)	0.15808 (10)	0.7333 (2)	0.0268 (7)	
C(12)	0.4476 (3)	0.20437 (11)	0.6829 (2)	0.0361 (9)	
C(13)	0.3800 (3)	0.15276 (11)	0.9010 (2)	0.0310 (8)	
C(14)	0.3031 (3)	0.11713 (12)	0.9350 (2)	0.0306 (8)	
C(15)	0.3344 (3)	0.10274 (13)	1.0327 (2)	0.0364 (9)	
C(16)	0.4424 (3)	0.12367 (13)	1.0950 (2)	0.0396 (9)	
C(17)	0.5179 (3)	0.15965 (13)	1.0630 (2)	0.0394 (9)	
C(18)	0.4864 (3)	0.17446 (12)	0.9666 (2)	0.0336 (8)	
C(19)	0.1179 (3)	0.06067 (12)	0.9001 (2)	0.0399 (10)	
C(20)	0.6557 (4)	0.23658 (15)	0.9951 (2)	0.0607 (13)	
C(21)	0.9136 (3)	0.10908 (10)	0.57963 (19)	0.0245 (7)	
C(22)	0.8872 (3)	0.13658 (10)	0.4944 (2)	0.0267 (7)	
C(23)	0.9542 (3)	0.12739 (10)	0.4183 (2)	0.0267 (7)	
C(24)	1.0484 (3)	0.08982 (10)	0.4250 (2)	0.0257 (7)	
C(25)	1.0758 (3)	0.06276 (11)	0.5098 (2)	0.0275 (8)	
C(26)	1.0083 (3)	0.07255 (10)	0.5861 (2)	0.0272 (7)	
C(27)	0.8261 (3)	0.18541 (12)	0.3144 (2)	0.0431 (10)	
C(28)	1.1946 (3)	0.04152 (11)	0.3470 (2)	0.0326 (8)	
C(29)	1.2407 (4)	0.03979 (13)	0.2500 (2)	0.0456 (10)	
C(30)	0.8354 (3)	0.11832 (10)	0.66079 (19)	0.0246 (7)	
C(31)	0.9029 (3)	0.15579 (10)	0.7346 (2)	0.0273 (7)	
C(32)	0.9155 (3)	0.20479 (11)	0.6913 (2)	0.0342 (9)	
C(33)	0.8574 (3)	0.14663 (11)	0.8994 (2)	0.0301 (8)	
C(34)	0.7920 (3)	0.10971 (12)	0.9378 (2)	0.0311 (8)	
C(35)	0.8316 (3)	0.09725 (14)	1.0365 (2)	0.0433 (10)	
C(36)	0.9362 (3)	0.12216 (15)	1.0940 (2)	0.0477 (11)	
C(37)	1.0023 (3)	0.15938 (15)	1.0574 (2)	0.0494 (11)	
C(38)	0.9632 (3)	0.17161 (12)	0.9596 (2)	0.0366 (9)	
C(39)	0.6114 (3)	0.05122 (12)	0.9101 (2)	0.0439 (10)	
C(40)	1.1206 (4)	0.23640 (16)	0.9716 (3)	0.0668 (14)	
H(2)	0.3186	0.1590	0.4841	0.031*	
H(5)	0.6376	0.0369	0.5173	0.031*	
H(6)	0.5278	0.0551	0.6446	0.030*	
H(15)	0.2826	0.0791	1.0553	0.045*	
H(16)	0.4646	0.1135	1.1594	0.048*	
H(17)	0.5897	0.1739	1.1059	0.047*	
H(22)	0.8237	0.1613	0.4891	0.032*	
H(25)	1.1393	0.0381	0.5156	0.033*	
H(26)	1.0273	0.0542	0.6425	0.033*	
H(35)	0.7879	0.0725	1.0626	0.053*	
H(36)	0.9629	0.1138	1.1592	0.057*	
H(37)	1.0721	0.1760	1.0976	0.059*	
H(10A)	0.2517	0.1348	0.6370	0.033*	
H(11A)	0.5124	0.1444	0.7652	0.033*	
H(30)	0.7429	0.1293	0.6318	0.030*	
H(31)	0.9930	0.1446	0.7682	0.033*	
H(8A)	0.7758	0.0454	0.4043	0.036*	
H(8B)	0.6536	0.0108	0.3602	0.036*	
H(12A)	0.3615	0.2216	0.6695	0.043*	
H(12B)	0.4760	0.1967	0.6221	0.043*	
H(28A)	1.2733	0.0447	0.3996	0.041*	
H(28B)	1.1448	0.0127	0.3563	0.040*	
H(32A)	0.9735	0.2025	0.6429	0.041*	
H(32B)	0.9570	0.2263	0.7428	0.041*	
H(7A)	0.2355	0.1658	0.2784	0.048*	
H(7B)	0.3409	0.2056	0.2648	0.048*	
H(7C)	0.3075	0.1959	0.3681	0.048*	
H(19A)	0.1582	0.0305	0.8892	0.049*	
H(19B)	0.0255	0.0619	0.8639	0.049*	
H(19C)	0.1172	0.0644	0.9682	0.049*	
H(20A)	0.6146	0.2630	1.0225	0.075*	
H(20B)	0.7239	0.2482	0.9605	0.075*	
H(20C)	0.6980	0.2156	1.0463	0.075*	
H(27A)	0.7432	0.1700	0.2827	0.053*	
H(27B)	0.8485	0.2106	0.2736	0.053*	
H(27C)	0.8130	0.1984	0.3754	0.052*	
H(39A)	0.6549	0.0209	0.9068	0.056*	
H(39B)	0.5204	0.0503	0.8717	0.055*	
H(39C)	0.6066	0.0582	0.9766	0.055*	
H(40A)	1.0798	0.2624	1.0002	0.081*	
H(40B)	1.1809	0.2486	0.9312	0.081*	
H(40C)	1.1720	0.2169	1.0221	0.081*	
H(9A)	0.6911	0.0188	0.2107	0.053*	
H(9B)	0.8418	0.0263	0.2672	0.053*	
H(9C)	0.7501	0.0705	0.2299	0.053*	
H(3)	0.3286	0.0846	0.7717	0.040*	
H(29A)	1.1769	0.0212	0.2048	0.058*	
H(29B)	1.3301	0.0255	0.2586	0.058*	
H(29C)	1.2446	0.0716	0.2253	0.058*	
H(4)	0.5138	0.2524	0.7713	0.058*	
H(10)	0.8214	0.0799	0.7677	0.038*	
H(11)	0.7260	0.2125	0.6741	0.057*	

### Atomic displacement parameters (Å^2^)

**Table:**

	*U*^11^	*U*^22^	*U*^33^	*U*^12^	*U*^13^	*U*^23^
O(1)	0.0501 (14)	0.0351 (12)	0.0190 (10)	0.0097 (10)	0.0106 (9)	0.0096 (8)
O(2)	0.0363 (13)	0.0358 (11)	0.0216 (10)	0.0068 (10)	0.0121 (9)	0.0019 (8)
O(3)	0.0427 (13)	0.0333 (11)	0.0228 (10)	−0.0069 (10)	0.0131 (9)	−0.0002 (8)
O(4)	0.0571 (17)	0.0383 (13)	0.0485 (14)	−0.0046 (12)	0.0134 (12)	−0.0073 (11)
O(5)	0.0382 (13)	0.0441 (13)	0.0178 (10)	0.0123 (10)	0.0100 (9)	0.0017 (9)
O(6)	0.0318 (12)	0.0519 (14)	0.0216 (10)	−0.0016 (10)	0.0081 (9)	0.0005 (9)
O(7)	0.0552 (17)	0.0563 (15)	0.0331 (12)	−0.0172 (13)	0.0115 (11)	−0.0087 (11)
O(8)	0.0493 (15)	0.0416 (13)	0.0234 (10)	0.0132 (11)	0.0139 (10)	0.0109 (9)
O(9)	0.0384 (13)	0.0334 (11)	0.0236 (10)	0.0044 (9)	0.0136 (9)	0.0017 (8)
O(10)	0.0415 (13)	0.0300 (11)	0.0224 (10)	−0.0044 (9)	0.0104 (9)	−0.0004 (8)
O(11)	0.0620 (18)	0.0404 (13)	0.0394 (13)	−0.0062 (12)	0.0077 (12)	0.0078 (10)
O(12)	0.0345 (12)	0.0377 (12)	0.0174 (9)	0.0038 (10)	0.0069 (8)	−0.0001 (8)
O(13)	0.0365 (13)	0.0475 (13)	0.0255 (11)	−0.0025 (10)	0.0117 (9)	0.0032 (9)
O(14)	0.0590 (17)	0.0576 (16)	0.0393 (14)	−0.0221 (14)	0.0111 (12)	−0.0151 (12)
C(1)	0.0261 (16)	0.0270 (15)	0.0198 (13)	−0.0055 (12)	0.0054 (11)	−0.0026 (11)
C(2)	0.0308 (17)	0.0258 (15)	0.0214 (14)	0.0031 (13)	0.0044 (12)	−0.0006 (11)
C(3)	0.0358 (18)	0.0248 (15)	0.0177 (13)	0.0012 (13)	0.0049 (12)	0.0016 (11)
C(4)	0.0244 (15)	0.0303 (15)	0.0191 (13)	−0.0027 (12)	0.0072 (11)	−0.0034 (11)
C(5)	0.0275 (16)	0.0271 (15)	0.0247 (14)	−0.0005 (13)	0.0067 (12)	0.0024 (11)
C(6)	0.0259 (16)	0.0296 (15)	0.0195 (13)	−0.0032 (13)	0.0021 (12)	0.0029 (11)
C(7)	0.056 (2)	0.0333 (17)	0.0290 (17)	0.0142 (16)	0.0112 (16)	0.0086 (14)
C(8)	0.0323 (18)	0.0249 (15)	0.0332 (16)	0.0015 (13)	0.0106 (14)	0.0002 (12)
C(9)	0.049 (2)	0.048 (2)	0.0348 (17)	0.0028 (17)	0.0235 (16)	−0.0029 (15)
C(10)	0.0289 (16)	0.0329 (16)	0.0199 (14)	−0.0009 (13)	0.0062 (12)	0.0012 (12)
C(11)	0.0300 (16)	0.0321 (16)	0.0194 (13)	0.0027 (13)	0.0076 (12)	0.0004 (12)
C(12)	0.052 (2)	0.0299 (16)	0.0259 (15)	−0.0069 (15)	0.0056 (15)	−0.0043 (13)
C(13)	0.0329 (18)	0.0421 (18)	0.0178 (13)	0.0096 (15)	0.0047 (12)	−0.0014 (12)
C(14)	0.0275 (17)	0.0443 (19)	0.0208 (14)	0.0047 (14)	0.0065 (12)	−0.0036 (13)
C(15)	0.0345 (18)	0.058 (2)	0.0200 (15)	0.0029 (16)	0.0121 (13)	0.0029 (14)
C(16)	0.044 (2)	0.057 (2)	0.0189 (15)	0.0063 (18)	0.0094 (14)	0.0012 (14)
C(17)	0.0332 (19)	0.060 (2)	0.0245 (16)	−0.0016 (17)	0.0039 (14)	−0.0100 (15)
C(18)	0.0365 (19)	0.0431 (19)	0.0243 (15)	−0.0003 (15)	0.0136 (13)	−0.0048 (13)
C(19)	0.036 (2)	0.050 (2)	0.0362 (18)	−0.0038 (16)	0.0124 (15)	−0.0040 (15)
C(20)	0.071 (3)	0.070 (2)	0.046 (2)	−0.028 (2)	0.024 (2)	−0.024 (2)
C(21)	0.0291 (16)	0.0282 (15)	0.0167 (13)	−0.0052 (13)	0.0060 (11)	−0.0021 (11)
C(22)	0.0296 (17)	0.0283 (15)	0.0227 (14)	0.0040 (13)	0.0066 (12)	0.0001 (12)
C(23)	0.0326 (17)	0.0297 (15)	0.0181 (13)	−0.0004 (13)	0.0056 (12)	0.0042 (11)
C(24)	0.0281 (16)	0.0303 (16)	0.0202 (14)	−0.0006 (13)	0.0086 (12)	−0.0015 (11)
C(25)	0.0274 (16)	0.0306 (16)	0.0259 (15)	0.0030 (13)	0.0083 (12)	0.0032 (12)
C(26)	0.0294 (17)	0.0321 (16)	0.0199 (14)	−0.0020 (13)	0.0041 (12)	0.0039 (12)
C(27)	0.055 (2)	0.044 (2)	0.0307 (17)	0.0150 (18)	0.0102 (16)	0.0121 (15)
C(28)	0.041 (2)	0.0267 (16)	0.0339 (17)	0.0018 (14)	0.0170 (15)	0.0001 (13)
C(29)	0.056 (2)	0.048 (2)	0.0412 (19)	0.0014 (18)	0.0292 (18)	−0.0037 (16)
C(30)	0.0278 (16)	0.0270 (15)	0.0197 (13)	−0.0041 (13)	0.0061 (12)	−0.0014 (11)
C(31)	0.0279 (17)	0.0344 (16)	0.0207 (14)	−0.0015 (13)	0.0077 (12)	−0.0029 (12)
C(32)	0.045 (2)	0.0308 (16)	0.0271 (16)	−0.0052 (15)	0.0072 (14)	−0.0020 (13)
C(33)	0.0283 (17)	0.0454 (19)	0.0174 (13)	0.0060 (14)	0.0065 (12)	−0.0045 (13)
C(34)	0.0270 (17)	0.0464 (19)	0.0210 (14)	0.0049 (14)	0.0072 (12)	−0.0036 (13)
C(35)	0.043 (2)	0.069 (2)	0.0204 (15)	0.0116 (19)	0.0134 (15)	0.0080 (15)
C(36)	0.047 (2)	0.080 (2)	0.0168 (15)	0.011 (2)	0.0063 (15)	−0.0012 (17)
C(37)	0.042 (2)	0.084 (2)	0.0226 (16)	0.002 (2)	0.0071 (15)	−0.0151 (18)
C(38)	0.0382 (19)	0.048 (2)	0.0244 (16)	0.0035 (16)	0.0074 (14)	−0.0080 (14)
C(39)	0.043 (2)	0.046 (2)	0.048 (2)	−0.0008 (17)	0.0241 (17)	0.0039 (17)
C(40)	0.057 (2)	0.075 (3)	0.071 (2)	−0.020 (2)	0.018 (2)	−0.036 (2)

### Geometric parameters (Å, °)

**Table:**

O(1)—C(3)	1.375 (3)	C(36)—C(37)	1.383 (5)
O(1)—C(7)	1.427 (4)	C(37)—C(38)	1.392 (4)
O(2)—C(4)	1.372 (3)	O(3)—H(3)	0.819
O(2)—C(8)	1.434 (3)	O(4)—H(4)	0.821
O(3)—C(10)	1.424 (3)	O(10)—H(10)	0.820
O(4)—C(12)	1.428 (3)	O(11)—H(11)	0.820
O(5)—C(11)	1.475 (3)	C(2)—H(2)	0.930
O(5)—C(13)	1.392 (3)	C(5)—H(5)	0.930
O(6)—C(14)	1.371 (3)	C(6)—H(6)	0.930
O(6)—C(19)	1.439 (4)	C(7)—H(7A)	0.960
O(7)—C(18)	1.377 (4)	C(7)—H(7B)	0.960
O(7)—C(20)	1.427 (4)	C(7)—H(7C)	0.960
O(8)—C(23)	1.381 (3)	C(8)—H(8A)	0.970
O(8)—C(27)	1.412 (4)	C(8)—H(8B)	0.970
O(9)—C(24)	1.371 (3)	C(9)—H(9A)	0.960
O(9)—C(28)	1.441 (3)	C(9)—H(9B)	0.960
O(10)—C(30)	1.429 (3)	C(9)—H(9C)	0.960
O(11)—C(32)	1.421 (4)	C(10)—H(10A)	0.980
O(12)—C(31)	1.469 (3)	C(11)—H(11A)	0.980
O(12)—C(33)	1.384 (3)	C(12)—H(12A)	0.970
O(13)—C(34)	1.376 (3)	C(12)—H(12B)	0.970
O(13)—C(39)	1.427 (4)	C(15)—H(15)	0.930
O(14)—C(38)	1.373 (4)	C(16)—H(16)	0.930
O(14)—C(40)	1.416 (4)	C(17)—H(17)	0.930
C(1)—C(2)	1.408 (3)	C(19)—H(19A)	0.960
C(1)—C(6)	1.380 (4)	C(19)—H(19B)	0.960
C(1)—C(10)	1.521 (4)	C(19)—H(19C)	0.960
C(2)—C(3)	1.381 (4)	C(20)—H(20A)	0.960
C(3)—C(4)	1.404 (4)	C(20)—H(20B)	0.960
C(4)—C(5)	1.391 (3)	C(20)—H(20C)	0.960
C(5)—C(6)	1.395 (4)	C(22)—H(22)	0.930
C(8)—C(9)	1.501 (4)	C(25)—H(25)	0.930
C(10)—C(11)	1.538 (3)	C(26)—H(26)	0.930
C(11)—C(12)	1.516 (4)	C(27)—H(27A)	0.960
C(13)—C(14)	1.395 (4)	C(27)—H(27B)	0.960
C(13)—C(18)	1.400 (4)	C(27)—H(27C)	0.960
C(14)—C(15)	1.402 (4)	C(28)—H(28A)	0.970
C(15)—C(16)	1.378 (4)	C(28)—H(28B)	0.970
C(16)—C(17)	1.383 (5)	C(29)—H(29A)	0.960
C(17)—C(18)	1.388 (4)	C(29)—H(29B)	0.960
C(21)—C(22)	1.402 (3)	C(29)—H(29C)	0.960
C(21)—C(26)	1.383 (4)	C(30)—H(30)	0.980
C(21)—C(30)	1.515 (4)	C(31)—H(31)	0.980
C(22)—C(23)	1.383 (4)	C(32)—H(32A)	0.970
C(23)—C(24)	1.401 (4)	C(32)—H(32B)	0.970
C(24)—C(25)	1.392 (3)	C(35)—H(35)	0.930
C(25)—C(26)	1.391 (4)	C(36)—H(36)	0.930
C(28)—C(29)	1.512 (5)	C(37)—H(37)	0.930
C(30)—C(31)	1.535 (3)	C(39)—H(39A)	0.960
C(31)—C(32)	1.518 (4)	C(39)—H(39B)	0.960
C(33)—C(34)	1.384 (4)	C(39)—H(39C)	0.960
C(33)—C(38)	1.403 (4)	C(40)—H(40A)	0.960
C(34)—C(35)	1.405 (4)	C(40)—H(40B)	0.960
C(35)—C(36)	1.377 (4)	C(40)—H(40C)	0.960
			
O(1)···C(16)^i^	3.335 (3)	H(36)···H(29C)^ix^	3.017
O(1)···C(29)^ii^	3.598 (4)	H(37)···O(4)^iv^	3.219
O(2)···C(22)	3.548 (3)	H(37)···O(11)^iv^	3.525
O(3)···C(8)^iii^	3.459 (3)	H(37)···C(7)^xv^	3.456
O(3)···C(9)^iii^	3.429 (4)	H(37)···C(15)^vi^	3.567
O(3)···C(25)^ii^	3.444 (3)	H(37)···H(15)^vi^	3.545
O(3)···C(26)^ii^	3.328 (3)	H(37)···H(7A)^xv^	2.747
O(4)···O(11)	2.882 (3)	H(37)···H(7B)^xv^	3.303
O(4)···O(12)	3.301 (3)	H(37)···H(4)^iv^	3.289
O(4)···C(7)^iv^	3.558 (4)	H(37)···H(11)^iv^	3.558
O(4)···C(27)^v^	3.475 (4)	H(10A)···C(21)^ii^	3.378
O(6)···C(31)^ii^	3.540 (3)	H(10A)···C(24)^ii^	3.491
O(6)···C(38)^ii^	3.513 (4)	H(10A)···C(25)^ii^	3.021
O(8)···C(36)^i^	3.422 (3)	H(10A)···C(26)^ii^	2.958
O(9)···C(2)^vi^	3.436 (3)	H(10A)···H(25)^ii^	3.284
O(9)···C(3)^vi^	3.569 (3)	H(10A)···H(26)^ii^	3.187
O(9)···C(7)^vi^	3.588 (4)	H(10A)···H(31)^ii^	3.440
O(10)···C(5)	3.387 (3)	H(10A)···H(32A)^ii^	3.367
O(10)···C(6)	3.283 (3)	H(10A)···H(20A)^viii^	3.437
O(10)···C(19)^vi^	3.570 (3)	H(11A)···O(12)	2.930
O(10)···C(28)^vii^	3.348 (3)	H(11A)···O(13)	2.636
O(10)···C(29)^vii^	3.336 (4)	H(11A)···C(33)	3.580
O(11)···O(4)	2.882 (3)	H(11A)···C(34)	3.455
O(11)···C(7)^iv^	3.479 (3)	H(11A)···C(39)	3.341
O(11)···C(12)	3.527 (4)	H(11A)···H(30)	3.242
O(11)···C(40)^viii^	2.903 (4)	H(11A)···H(39B)	3.025
O(12)···O(4)	3.301 (3)	H(11A)···H(10)	3.556
O(13)···C(11)	3.572 (3)	H(11A)···H(11)	3.286
O(13)···C(18)	3.571 (4)	H(30)···C(1)	3.265
C(1)···C(25)^ii^	3.553 (4)	H(30)···C(4)	3.390
C(2)···O(9)^ii^	3.436 (3)	H(30)···C(5)	2.866
C(2)···C(24)^ii^	3.517 (4)	H(30)···C(6)	2.794
C(3)···O(9)^ii^	3.569 (3)	H(30)···H(5)	3.122
C(3)···C(28)^ii^	3.442 (4)	H(30)···H(6)	3.013
C(5)···O(10)	3.387 (3)	H(30)···H(11A)	3.242
C(5)···C(21)	3.582 (4)	H(30)···H(12B)	3.238
C(5)···C(30)	3.407 (3)	H(31)···O(5)^vi^	3.463
C(6)···O(10)	3.283 (3)	H(31)···O(6)^vi^	2.580
C(6)···C(30)	3.465 (4)	H(31)···C(14)^vi^	3.576
C(7)···O(4)^viii^	3.558 (4)	H(31)···C(19)^vi^	3.101
C(7)···O(9)^ii^	3.588 (4)	H(31)···H(10A)^vi^	3.440
C(7)···O(11)^viii^	3.479 (3)	H(31)···H(19B)^vi^	2.667
C(8)···O(3)^iii^	3.459 (3)	H(8A)···C(21)	3.132
C(8)···C(23)	3.513 (4)	H(8A)···C(22)	2.973
C(9)···O(3)^iii^	3.429 (4)	H(8A)···C(23)	2.889
C(10)···C(25)^ii^	3.492 (3)	H(8A)···C(24)	2.944
C(10)···C(26)^ii^	3.566 (4)	H(8A)···C(25)	3.101
C(11)···O(13)	3.572 (3)	H(8A)···C(25)^vii^	3.486
C(12)···O(11)	3.527 (4)	H(8A)···C(26)	3.186
C(16)···O(1)^ix^	3.335 (3)	H(8A)···H(22)	3.464
C(18)···O(13)	3.571 (4)	H(8A)···H(25)^vii^	2.663
C(19)···O(10)^ii^	3.570 (3)	H(8A)···H(26)^vii^	3.542
C(19)···C(33)^ii^	3.536 (4)	H(8B)···O(3)^iii^	2.738
C(21)···C(5)	3.582 (4)	H(8B)···C(1)^iii^	3.562
C(22)···O(2)	3.548 (3)	H(8B)···C(6)^iii^	3.006
C(23)···C(8)	3.513 (4)	H(8B)···C(25)^vii^	3.596
C(24)···C(2)^vi^	3.517 (4)	H(8B)···H(6)^iii^	2.573
C(25)···O(3)^vi^	3.444 (3)	H(8B)···H(25)^vii^	2.787
C(25)···C(1)^vi^	3.553 (4)	H(8B)···H(3)^iii^	3.275
C(25)···C(10)^vi^	3.492 (3)	H(8B)···H(29B)^ii^	3.283
C(26)···O(3)^vi^	3.328 (3)	H(12A)···C(20)^viii^	3.100
C(26)···C(10)^vi^	3.566 (4)	H(12A)···C(27)^v^	3.362
C(27)···O(4)^x^	3.475 (4)	H(12A)···H(20A)^viii^	2.917
C(28)···O(10)^vii^	3.348 (3)	H(12A)···H(20B)^viii^	3.100
C(28)···C(3)^vi^	3.442 (4)	H(12A)···H(20C)^viii^	2.764
C(29)···O(1)^vi^	3.598 (4)	H(12A)···H(27B)^v^	2.414
C(29)···O(10)^vii^	3.336 (4)	H(12A)···H(40A)^viii^	3.529
C(30)···C(5)	3.407 (3)	H(12B)···O(11)	3.115
C(30)···C(6)	3.465 (4)	H(12B)···C(40)^viii^	3.341
C(31)···O(6)^vi^	3.540 (3)	H(12B)···H(30)	3.238
C(33)···C(19)^vi^	3.536 (4)	H(12B)···H(20B)^viii^	3.408
C(36)···O(8)^ix^	3.422 (3)	H(12B)···H(40A)^viii^	2.438
C(38)···O(6)^vi^	3.513 (4)	H(12B)···H(40C)^viii^	3.556
C(40)···O(11)^iv^	2.903 (4)	H(12B)···H(11)	2.490
O(1)···H(16)^i^	2.614	H(28A)···O(1)^vi^	3.506
O(1)···H(28A)^ii^	3.506	H(28A)···C(1)^vi^	3.189
O(1)···H(27A)	3.367	H(28A)···C(2)^vi^	2.950
O(1)···H(29C)^ii^	2.975	H(28A)···C(3)^vi^	2.829
O(2)···H(16)^i^	2.862	H(28A)···C(4)^vi^	2.918
O(2)···H(22)	3.523	H(28A)···C(5)^vi^	3.129
O(2)···H(27A)	3.090	H(28A)···C(5)^vii^	3.471
O(2)···H(29B)^ii^	3.152	H(28A)···C(6)^vi^	3.256
O(3)···H(25)^ii^	3.241	H(28A)···H(2)^vi^	3.419
O(3)···H(26)^ii^	3.030	H(28A)···H(5)^vii^	2.643
O(3)···H(8B)^iii^	2.738	H(28A)···H(6)^vii^	3.547
O(3)···H(39B)	2.753	H(28B)···O(10)^vii^	2.656
O(3)···H(9A)^iii^	2.949	H(28B)···C(26)^vii^	3.025
O(3)···H(9B)^iii^	3.423	H(28B)···H(5)^vii^	2.877
O(4)···H(37)^viii^	3.219	H(28B)···H(26)^vii^	2.541
O(4)···H(7A)^iv^	3.339	H(28B)···H(9B)	3.053
O(4)···H(7B)^iv^	3.299	H(28B)···H(10)^vii^	3.176
O(4)···H(7C)^iv^	3.451	H(32A)···C(20)^x^	3.448
O(4)···H(27B)^v^	2.652	H(32A)···H(10A)^vi^	3.367
O(4)···H(40A)^viii^	3.388	H(32A)···H(7B)^iv^	3.483
O(4)···H(11)	2.191	H(32A)···H(20A)^x^	2.576
O(5)···H(31)^ii^	3.463	H(32B)···C(7)^iv^	3.143
O(5)···H(27B)^v^	3.389	H(32B)···H(7B)^iv^	2.282
O(5)···H(40B)^ii^	3.390	H(32B)···H(7C)^iv^	3.322
O(6)···H(26)^ii^	3.512	H(7A)···O(4)^viii^	3.339
O(6)···H(31)^ii^	2.580	H(7A)···O(8)^ii^	3.240
O(6)···H(39B)	3.478	H(7A)···O(9)^ii^	2.905
O(7)···H(27B)^v^	3.464	H(7A)···C(29)^ii^	3.560
O(7)···H(27C)^v^	3.462	H(7A)···C(37)^xi^	3.496
O(8)···H(36)^i^	2.694	H(7A)···H(16)^i^	3.397
O(8)···H(7A)^vi^	3.240	H(7A)···H(36)^xi^	3.243
O(8)···H(9C)	3.092	H(7A)···H(37)^xi^	2.747
O(8)···H(4)^x^	2.966	H(7A)···H(20B)^viii^	3.527
O(9)···H(2)^vi^	3.363	H(7A)···H(29C)^ii^	2.753
O(9)···H(36)^i^	2.851	H(7A)···H(4)^viii^	3.169
O(9)···H(7A)^vi^	2.905	H(7B)···O(4)^viii^	3.299
O(9)···H(9B)	3.076	H(7B)···O(11)^viii^	2.589
O(10)···H(5)	3.152	H(7B)···O(14)^viii^	3.437
O(10)···H(6)	2.962	H(7B)···C(16)^i^	3.585
O(10)···H(28B)^vii^	2.656	H(7B)···C(32)^viii^	2.867
O(10)···H(19B)^vi^	2.663	H(7B)···H(16)^i^	3.325
O(10)···H(29A)^vii^	2.935	H(7B)···H(37)^xi^	3.303
O(10)···H(29B)^vii^	3.264	H(7B)···H(32A)^viii^	3.483
O(11)···H(37)^viii^	3.525	H(7B)···H(32B)^viii^	2.282
O(11)···H(12B)	3.115	H(7B)···H(20B)^viii^	3.426
O(11)···H(7B)^iv^	2.589	H(7B)···H(40C)^xi^	3.503
O(11)···H(40A)^viii^	2.632	H(7B)···H(4)^viii^	3.473
O(11)···H(40B)^viii^	3.094	H(7B)···H(11)^viii^	2.764
O(11)···H(40C)^viii^	2.526	H(7C)···O(4)^viii^	3.451
O(11)···H(4)	3.577	H(7C)···O(14)^viii^	3.375
O(12)···H(11A)	2.930	H(7C)···C(20)^viii^	3.168
O(12)···H(19B)^vi^	3.501	H(7C)···H(32B)^viii^	3.322
O(12)···H(20B)	3.507	H(7C)···H(20A)^viii^	3.352
O(13)···H(6)	3.419	H(7C)···H(20B)^viii^	2.288
O(13)···H(11A)	2.636	H(7C)···H(40A)^viii^	3.192
O(13)···H(19B)^vi^	3.439	H(7C)···H(4)^viii^	3.309
O(13)···H(3)	3.596	H(19A)···C(9)^iii^	3.075
O(13)···H(29A)^vii^	3.574	H(19A)···H(26)^ii^	3.512
O(14)···H(7B)^iv^	3.437	H(19A)···H(35)^xii^	2.994
O(14)···H(7C)^iv^	3.375	H(19A)···H(39A)^xii^	3.414
O(14)···H(20B)	3.285	H(19A)···H(9A)^iii^	2.629
C(1)···H(25)^ii^	3.353	H(19A)···H(9B)^iii^	2.707
C(1)···H(30)	3.265	H(19A)···H(9C)^iii^	3.496
C(1)···H(8B)^iii^	3.562	H(19B)···O(10)^ii^	2.663
C(1)···H(28A)^ii^	3.189	H(19B)···O(12)^ii^	3.501
C(2)···H(28A)^ii^	2.950	H(19B)···O(13)^ii^	3.439
C(2)···H(40A)^viii^	3.469	H(19B)···C(30)^ii^	3.479
C(3)···H(28A)^ii^	2.829	H(19B)···C(31)^ii^	3.295
C(3)···H(40A)^viii^	3.542	H(19B)···C(33)^ii^	3.001
C(3)···H(29B)^ii^	3.600	H(19B)···C(34)^ii^	3.026
C(3)···H(29C)^ii^	3.371	H(19B)···C(35)^ii^	3.505
C(4)···H(22)	3.434	H(19B)···C(38)^ii^	3.460
C(4)···H(30)	3.390	H(19B)···H(26)^ii^	3.107
C(4)···H(28A)^ii^	2.918	H(19B)···H(31)^ii^	2.667
C(4)···H(29B)^ii^	3.328	H(19B)···H(9B)^iii^	3.485
C(5)···H(5)^iii^	3.450	H(19B)···H(29A)^iii^	3.110
C(5)···H(30)	2.866	H(19B)···H(10)^ii^	2.267
C(5)···H(28A)^ii^	3.129	H(19C)···C(33)^ii^	3.460
C(5)···H(28A)^vii^	3.471	H(19C)···C(34)^ii^	3.422
C(6)···H(5)^iii^	3.586	H(19C)···C(35)^ii^	3.292
C(6)···H(30)	2.794	H(19C)···C(36)^ii^	3.188
C(6)···H(8B)^iii^	3.006	H(19C)···C(37)^ii^	3.242
C(6)···H(28A)^ii^	3.256	H(19C)···C(38)^ii^	3.368
C(7)···H(16)^i^	3.345	H(19C)···H(39A)^xii^	3.521
C(7)···H(37)^xi^	3.456	H(19C)···H(29A)^xiii^	3.467
C(7)···H(32B)^viii^	3.143	H(19C)···H(29C)^xiii^	3.583
C(7)···H(20B)^viii^	3.180	H(20A)···C(22)^v^	3.586
C(7)···H(29C)^ii^	3.327	H(20A)···C(32)^v^	3.475
C(7)···H(4)^viii^	3.507	H(20A)···H(2)^iv^	3.099
C(7)···H(11)^viii^	3.543	H(20A)···H(22)^v^	3.545
C(8)···H(6)^iii^	3.512	H(20A)···H(10A)^iv^	3.437
C(8)···H(25)^vii^	3.128	H(20A)···H(12A)^iv^	2.917
C(9)···H(16)^i^	3.577	H(20A)···H(32A)^v^	2.576
C(9)···H(26)^vii^	3.540	H(20A)···H(7C)^iv^	3.352
C(9)···H(35)^i^	2.957	H(20A)···H(27C)^v^	3.468
C(9)···H(36)^i^	3.436	H(20B)···O(12)	3.507
C(9)···H(19A)^iii^	3.075	H(20B)···O(14)	3.285
C(9)···H(3)^iii^	3.558	H(20B)···C(7)^iv^	3.180
C(10)···H(25)^ii^	3.501	H(20B)···C(33)	3.323
C(11)···H(11)	3.596	H(20B)···C(38)	3.206
C(12)···H(27B)^v^	2.958	H(20B)···H(2)^iv^	2.769
C(12)···H(40A)^viii^	3.224	H(20B)···H(12A)^iv^	3.100
C(12)···H(11)	2.800	H(20B)···H(12B)^iv^	3.408
C(13)···H(39B)	3.255	H(20B)···H(7A)^iv^	3.527
C(13)···H(39C)	3.511	H(20B)···H(7B)^iv^	3.426
C(13)···H(40B)^ii^	3.412	H(20B)···H(7C)^iv^	2.288
C(13)···H(40C)^ii^	3.421	H(20B)···H(40A)	3.494
C(14)···H(31)^ii^	3.576	H(20C)···C(33)	3.423
C(14)···H(39B)	3.112	H(20C)···C(34)	3.543
C(14)···H(39C)	3.390	H(20C)···C(35)	3.590
C(14)···H(40C)^ii^	3.410	H(20C)···C(36)	3.510
C(15)···H(37)^ii^	3.567	H(20C)···C(37)	3.387
C(15)···H(39A)^xii^	3.568	H(20C)···C(38)	3.339
C(15)···H(39B)	3.497	H(20C)···H(12A)^iv^	2.764
C(15)···H(39C)	3.208	H(20C)···H(27A)^ix^	3.494
C(15)···H(40C)^ii^	3.578	H(20C)···H(27B)^ix^	3.248
C(15)···H(29C)^xiii^	3.119	H(27A)···O(1)	3.367
C(16)···H(7B)^ix^	3.585	H(27A)···O(2)	3.090
C(16)···H(39C)	3.132	H(27A)···C(17)^i^	3.452
C(16)···H(9C)^ix^	3.596	H(27A)···H(16)^i^	3.366
C(16)···H(29C)^xiii^	3.267	H(27A)···H(17)^i^	2.649
C(17)···H(27A)^ix^	3.452	H(27A)···H(36)^i^	3.413
C(17)···H(39C)	3.279	H(27A)···H(20C)^i^	3.494
C(18)···H(39C)	3.469	H(27A)···H(40B)^viii^	3.226
C(18)···H(40C)^ii^	3.562	H(27A)···H(9C)	2.892
C(19)···H(26)^ii^	3.547	H(27A)···H(4)^x^	3.485
C(19)···H(31)^ii^	3.101	H(27B)···O(4)^x^	2.652
C(19)···H(9A)^iii^	3.478	H(27B)···O(5)^x^	3.389
C(19)···H(9B)^iii^	3.456	H(27B)···O(7)^x^	3.464
C(19)···H(10)^ii^	3.212	H(27B)···C(12)^x^	2.958
C(20)···H(2)^iv^	3.366	H(27B)···H(17)^i^	3.298
C(20)···H(12A)^iv^	3.100	H(27B)···H(36)^i^	3.452
C(20)···H(32A)^v^	3.448	H(27B)···H(12A)^x^	2.414
C(20)···H(7C)^iv^	3.168	H(27B)···H(20C)^i^	3.248
C(21)···H(5)	3.386	H(27B)···H(40B)^viii^	3.214
C(21)···H(10A)^vi^	3.378	H(27B)···H(4)^x^	1.947
C(21)···H(8A)	3.132	H(27C)···O(7)^x^	3.462
C(22)···H(8A)	2.973	H(27C)···C(40)^viii^	3.127
C(22)···H(20A)^x^	3.586	H(27C)···H(20A)^x^	3.468
C(23)···H(8A)	2.889	H(27C)···H(40A)^viii^	3.340
C(23)···H(9B)	3.586	H(27C)···H(40B)^viii^	2.220
C(23)···H(9C)	3.401	H(27C)···H(40C)^viii^	3.593
C(24)···H(2)^vi^	3.288	H(27C)···H(4)^x^	3.016
C(24)···H(10A)^vi^	3.491	H(39A)···C(15)^xii^	3.568
C(24)···H(8A)	2.944	H(39A)···C(29)^vii^	3.106
C(24)···H(9B)	3.245	H(39A)···H(15)^xii^	2.902
C(25)···H(25)^vii^	3.522	H(39A)···H(19A)^xii^	3.414
C(25)···H(10A)^vi^	3.021	H(39A)···H(19C)^xii^	3.521
C(25)···H(8A)	3.101	H(39A)···H(29A)^vii^	2.758
C(25)···H(8A)^vii^	3.486	H(39A)···H(29B)^vii^	2.682
C(25)···H(8B)^vii^	3.596	H(39A)···H(29C)^vii^	3.441
C(26)···H(10A)^vi^	2.958	H(39B)···O(3)	2.753
C(26)···H(8A)	3.186	H(39B)···O(6)	3.478
C(26)···H(28B)^vii^	3.025	H(39B)···C(13)	3.255
C(27)···H(17)^i^	3.393	H(39B)···C(14)	3.112
C(27)···H(36)^i^	3.422	H(39B)···C(15)	3.497
C(27)···H(12A)^x^	3.362	H(39B)···H(6)	3.192
C(27)···H(40B)^viii^	3.012	H(39B)···H(11A)	3.025
C(27)···H(9C)	3.468	H(39B)···H(9A)^iii^	2.931
C(27)···H(4)^x^	2.703	H(39B)···H(3)	2.344
C(28)···H(5)^vii^	3.165	H(39B)···H(29B)^vii^	3.328
C(28)···H(26)^vii^	3.495	H(39C)···C(13)	3.511
C(28)···H(9B)	3.495	H(39C)···C(14)	3.390
C(29)···H(15)^xiv^	3.039	H(39C)···C(15)	3.208
C(29)···H(16)^xiv^	3.449	H(39C)···C(16)	3.132
C(29)···H(36)^i^	3.494	H(39C)···C(17)	3.279
C(29)···H(7A)^vi^	3.560	H(39C)···C(18)	3.469
C(29)···H(39A)^vii^	3.106	H(39C)···H(16)	3.507
C(29)···H(10)^vii^	3.415	H(39C)···H(9A)^ix^	3.406
C(30)···H(5)	3.407	H(39C)···H(9C)^ix^	3.575
C(30)···H(6)	3.502	H(40A)···O(4)^iv^	3.388
C(30)···H(19B)^vi^	3.479	H(40A)···O(11)^iv^	2.632
C(31)···H(19B)^vi^	3.295	H(40A)···C(2)^iv^	3.469
C(32)···H(7B)^iv^	2.867	H(40A)···C(3)^iv^	3.542
C(32)···H(20A)^x^	3.475	H(40A)···C(12)^iv^	3.224
C(33)···H(11A)	3.580	H(40A)···H(2)^iv^	3.379
C(33)···H(19B)^vi^	3.001	H(40A)···H(22)^iv^	3.259
C(33)···H(19C)^vi^	3.460	H(40A)···H(12A)^iv^	3.529
C(33)···H(20B)	3.323	H(40A)···H(12B)^iv^	2.438
C(33)···H(20C)	3.423	H(40A)···H(7C)^iv^	3.192
C(34)···H(11A)	3.455	H(40A)···H(20B)	3.494
C(34)···H(19B)^vi^	3.026	H(40A)···H(27C)^iv^	3.340
C(34)···H(19C)^vi^	3.422	H(40A)···H(11)^iv^	2.679
C(34)···H(20C)	3.543	H(40B)···O(5)^vi^	3.390
C(35)···H(17)	3.497	H(40B)···O(11)^iv^	3.094
C(35)···H(19B)^vi^	3.505	H(40B)···C(13)^vi^	3.412
C(35)···H(19C)^vi^	3.292	H(40B)···C(27)^iv^	3.012
C(35)···H(20C)	3.590	H(40B)···H(22)^iv^	2.937
C(35)···H(9C)^ix^	3.061	H(40B)···H(27A)^iv^	3.226
C(36)···H(19C)^vi^	3.188	H(40B)···H(27B)^iv^	3.214
C(36)···H(20C)	3.510	H(40B)···H(27C)^iv^	2.220
C(36)···H(9C)^ix^	3.235	H(40B)···H(11)^iv^	3.514
C(36)···H(29C)^ix^	3.553	H(40C)···O(11)^iv^	2.526
C(37)···H(15)^vi^	3.584	H(40C)···C(13)^vi^	3.421
C(37)···H(7A)^xv^	3.496	H(40C)···C(14)^vi^	3.410
C(37)···H(19C)^vi^	3.242	H(40C)···C(15)^vi^	3.578
C(37)···H(20C)	3.387	H(40C)···C(18)^vi^	3.562
C(38)···H(19B)^vi^	3.460	H(40C)···H(12B)^iv^	3.556
C(38)···H(19C)^vi^	3.368	H(40C)···H(7B)^xv^	3.503
C(38)···H(20B)	3.206	H(40C)···H(27C)^iv^	3.593
C(38)···H(20C)	3.339	H(40C)···H(11)^iv^	2.880
C(39)···H(11A)	3.341	H(9A)···O(3)^iii^	2.949
C(39)···H(3)	3.232	H(9A)···C(19)^iii^	3.478
C(39)···H(29A)^vii^	3.523	H(9A)···H(16)^i^	3.466
C(39)···H(29B)^vii^	3.326	H(9A)···H(35)^i^	2.871
C(40)···H(22)^iv^	3.491	H(9A)···H(19A)^iii^	2.629
C(40)···H(12B)^iv^	3.341	H(9A)···H(39B)^iii^	2.931
C(40)···H(27C)^iv^	3.127	H(9A)···H(39C)^i^	3.406
C(40)···H(11)^iv^	3.170	H(9A)···H(3)^iii^	2.923
H(2)···O(9)^ii^	3.363	H(9B)···O(3)^iii^	3.423
H(2)···C(20)^viii^	3.366	H(9B)···O(9)	3.076
H(2)···C(24)^ii^	3.288	H(9B)···C(19)^iii^	3.456
H(2)···H(28A)^ii^	3.419	H(9B)···C(23)	3.586
H(2)···H(20A)^viii^	3.099	H(9B)···C(24)	3.245
H(2)···H(20B)^viii^	2.769	H(9B)···C(28)	3.495
H(2)···H(40A)^viii^	3.379	H(9B)···H(25)^vii^	3.509
H(5)···O(10)	3.152	H(9B)···H(26)^vii^	2.788
H(5)···C(5)^iii^	3.450	H(9B)···H(35)^i^	3.094
H(5)···C(6)^iii^	3.586	H(9B)···H(36)^i^	3.232
H(5)···C(21)	3.386	H(9B)···H(28B)	3.053
H(5)···C(28)^vii^	3.165	H(9B)···H(19A)^iii^	2.707
H(5)···C(30)	3.407	H(9B)···H(19B)^iii^	3.485
H(5)···H(5)^iii^	3.389	H(9B)···H(3)^iii^	3.536
H(5)···H(25)^vii^	3.151	H(9B)···H(29A)	3.600
H(5)···H(30)	3.122	H(9C)···O(8)	3.092
H(5)···H(28A)^vii^	2.643	H(9C)···C(16)^i^	3.596
H(5)···H(28B)^vii^	2.877	H(9C)···C(23)	3.401
H(5)···H(29B)^vii^	3.552	H(9C)···C(27)	3.468
H(6)···O(10)	2.962	H(9C)···C(35)^i^	3.061
H(6)···O(13)	3.419	H(9C)···C(36)^i^	3.235
H(6)···C(8)^iii^	3.512	H(9C)···H(16)^i^	3.067
H(6)···C(30)	3.502	H(9C)···H(17)^i^	3.593
H(6)···H(30)	3.013	H(9C)···H(35)^i^	2.441
H(6)···H(8B)^iii^	2.573	H(9C)···H(36)^i^	2.777
H(6)···H(28A)^vii^	3.547	H(9C)···H(19A)^iii^	3.496
H(6)···H(39B)	3.192	H(9C)···H(27A)	2.892
H(6)···H(29B)^vii^	2.865	H(9C)···H(39C)^i^	3.575
H(6)···H(10)	3.168	H(3)···O(13)	3.596
H(15)···C(29)^xiii^	3.039	H(3)···C(9)^iii^	3.558
H(15)···C(37)^ii^	3.584	H(3)···C(39)	3.232
H(15)···H(37)^ii^	3.545	H(3)···H(26)^ii^	3.300
H(15)···H(39A)^xii^	2.902	H(3)···H(8B)^iii^	3.275
H(15)···H(29A)^xiii^	2.991	H(3)···H(39B)	2.344
H(15)···H(29B)^xiii^	3.171	H(3)···H(9A)^iii^	2.923
H(15)···H(29C)^xiii^	2.486	H(3)···H(9B)^iii^	3.536
H(16)···O(1)^ix^	2.614	H(29A)···O(10)^vii^	2.935
H(16)···O(2)^ix^	2.862	H(29A)···O(13)^vii^	3.574
H(16)···C(7)^ix^	3.345	H(29A)···C(39)^vii^	3.523
H(16)···C(9)^ix^	3.577	H(29A)···H(15)^xiv^	2.991
H(16)···C(29)^xiii^	3.449	H(29A)···H(36)^i^	3.341
H(16)···H(7A)^ix^	3.397	H(29A)···H(19B)^iii^	3.110
H(16)···H(7B)^ix^	3.325	H(29A)···H(19C)^xiv^	3.467
H(16)···H(27A)^ix^	3.366	H(29A)···H(39A)^vii^	2.758
H(16)···H(39C)	3.507	H(29A)···H(9B)	3.600
H(16)···H(9A)^ix^	3.466	H(29A)···H(10)^vii^	2.863
H(16)···H(9C)^ix^	3.067	H(29B)···O(2)^vi^	3.152
H(16)···H(29B)^xiii^	3.241	H(29B)···O(10)^vii^	3.264
H(16)···H(29C)^xiii^	2.788	H(29B)···C(3)^vi^	3.600
H(17)···C(27)^ix^	3.393	H(29B)···C(4)^vi^	3.328
H(17)···C(35)	3.497	H(29B)···C(39)^vii^	3.326
H(17)···H(35)	3.577	H(29B)···H(5)^vii^	3.552
H(17)···H(27A)^ix^	2.649	H(29B)···H(6)^vii^	2.865
H(17)···H(27B)^ix^	3.298	H(29B)···H(15)^xiv^	3.171
H(17)···H(9C)^ix^	3.593	H(29B)···H(16)^xiv^	3.241
H(22)···O(2)	3.523	H(29B)···H(8B)^vi^	3.283
H(22)···C(4)	3.434	H(29B)···H(39A)^vii^	2.682
H(22)···C(40)^viii^	3.491	H(29B)···H(39B)^vii^	3.328
H(22)···H(8A)	3.464	H(29B)···H(10)^vii^	3.306
H(22)···H(20A)^x^	3.545	H(29C)···O(1)^vi^	2.975
H(22)···H(40A)^viii^	3.259	H(29C)···C(3)^vi^	3.371
H(22)···H(40B)^viii^	2.937	H(29C)···C(7)^vi^	3.327
H(25)···O(3)^vi^	3.241	H(29C)···C(15)^xiv^	3.119
H(25)···C(1)^vi^	3.353	H(29C)···C(16)^xiv^	3.267
H(25)···C(8)^vii^	3.128	H(29C)···C(36)^i^	3.553
H(25)···C(10)^vi^	3.501	H(29C)···H(15)^xiv^	2.486
H(25)···C(25)^vii^	3.522	H(29C)···H(16)^xiv^	2.788
H(25)···H(5)^vii^	3.151	H(29C)···H(36)^i^	3.017
H(25)···H(25)^vii^	3.456	H(29C)···H(7A)^vi^	2.753
H(25)···H(26)^vii^	3.597	H(29C)···H(19C)^xiv^	3.583
H(25)···H(10A)^vi^	3.284	H(29C)···H(39A)^vii^	3.441
H(25)···H(8A)^vii^	2.663	H(4)···O(8)^v^	2.966
H(25)···H(8B)^vii^	2.787	H(4)···O(11)	3.577
H(25)···H(9B)^vii^	3.509	H(4)···C(7)^iv^	3.507
H(26)···O(3)^vi^	3.030	H(4)···C(27)^v^	2.703
H(26)···O(6)^vi^	3.512	H(4)···H(37)^viii^	3.289
H(26)···C(9)^vii^	3.540	H(4)···H(7A)^iv^	3.169
H(26)···C(19)^vi^	3.547	H(4)···H(7B)^iv^	3.473
H(26)···C(28)^vii^	3.495	H(4)···H(7C)^iv^	3.309
H(26)···H(25)^vii^	3.597	H(4)···H(27A)^v^	3.485
H(26)···H(10A)^vi^	3.187	H(4)···H(27B)^v^	1.947
H(26)···H(8A)^vii^	3.542	H(4)···H(27C)^v^	3.016
H(26)···H(28B)^vii^	2.541	H(4)···H(11)	2.938
H(26)···H(19A)^vi^	3.512	H(10)···C(19)^vi^	3.212
H(26)···H(19B)^vi^	3.107	H(10)···C(29)^vii^	3.415
H(26)···H(9B)^vii^	2.788	H(10)···H(6)	3.168
H(26)···H(3)^vi^	3.300	H(10)···H(11A)	3.556
H(35)···C(9)^ix^	2.957	H(10)···H(28B)^vii^	3.176
H(35)···H(17)	3.577	H(10)···H(19B)^vi^	2.267
H(35)···H(19A)^xii^	2.994	H(10)···H(29A)^vii^	2.863
H(35)···H(9A)^ix^	2.871	H(10)···H(29B)^vii^	3.306
H(35)···H(9B)^ix^	3.094	H(11)···O(4)	2.191
H(35)···H(9C)^ix^	2.441	H(11)···C(7)^iv^	3.543
H(36)···O(8)^ix^	2.694	H(11)···C(11)	3.596
H(36)···O(9)^ix^	2.851	H(11)···C(12)	2.800
H(36)···C(9)^ix^	3.436	H(11)···C(40)^viii^	3.170
H(36)···C(27)^ix^	3.422	H(11)···H(37)^viii^	3.558
H(36)···C(29)^ix^	3.494	H(11)···H(11A)	3.286
H(36)···H(7A)^xv^	3.243	H(11)···H(12B)	2.490
H(36)···H(27A)^ix^	3.413	H(11)···H(7B)^iv^	2.764
H(36)···H(27B)^ix^	3.452	H(11)···H(40A)^viii^	2.679
H(36)···H(9B)^ix^	3.232	H(11)···H(40B)^viii^	3.514
H(36)···H(9C)^ix^	2.777	H(11)···H(40C)^viii^	2.880
H(36)···H(29A)^ix^	3.341	H(11)···H(4)	2.938
			
C(3)—O(1)—C(7)	117.5 (2)	O(2)—C(8)—H(8B)	110.0
C(4)—O(2)—C(8)	117.0 (2)	C(9)—C(8)—H(8A)	110.0
C(11)—O(5)—C(13)	119.1 (2)	C(9)—C(8)—H(8B)	110.1
C(14)—O(6)—C(19)	117.8 (2)	H(8A)—C(8)—H(8B)	109.5
C(18)—O(7)—C(20)	117.9 (2)	C(8)—C(9)—H(9A)	109.4
C(23)—O(8)—C(27)	117.1 (2)	C(8)—C(9)—H(9B)	109.5
C(24)—O(9)—C(28)	116.8 (2)	C(8)—C(9)—H(9C)	109.4
C(31)—O(12)—C(33)	117.9 (2)	H(9A)—C(9)—H(9B)	109.5
C(34)—O(13)—C(39)	118.4 (2)	H(9A)—C(9)—H(9C)	109.5
C(38)—O(14)—C(40)	117.4 (2)	H(9B)—C(9)—H(9C)	109.5
C(2)—C(1)—C(6)	118.5 (2)	O(3)—C(10)—H(10A)	109.0
C(2)—C(1)—C(10)	120.4 (2)	C(1)—C(10)—H(10A)	109.0
C(6)—C(1)—C(10)	121.0 (2)	C(11)—C(10)—H(10A)	109.0
C(1)—C(2)—C(3)	121.1 (2)	O(5)—C(11)—H(11A)	110.3
O(1)—C(3)—C(2)	125.4 (2)	C(10)—C(11)—H(11A)	110.3
O(1)—C(3)—C(4)	114.6 (2)	C(12)—C(11)—H(11A)	110.2
C(2)—C(3)—C(4)	119.9 (2)	O(4)—C(12)—H(12A)	108.8
O(2)—C(4)—C(3)	115.9 (2)	O(4)—C(12)—H(12B)	107.6
O(2)—C(4)—C(5)	125.0 (2)	C(11)—C(12)—H(12A)	108.2
C(3)—C(4)—C(5)	119.1 (2)	C(11)—C(12)—H(12B)	108.3
C(4)—C(5)—C(6)	120.4 (2)	H(12A)—C(12)—H(12B)	109.5
C(1)—C(6)—C(5)	120.9 (2)	C(14)—C(15)—H(15)	120.1
O(2)—C(8)—C(9)	107.2 (2)	C(16)—C(15)—H(15)	120.2
O(3)—C(10)—C(1)	107.5 (2)	C(15)—C(16)—H(16)	119.6
O(3)—C(10)—C(11)	109.9 (2)	C(17)—C(16)—H(16)	119.7
C(1)—C(10)—C(11)	112.4 (2)	C(16)—C(17)—H(17)	120.0
O(5)—C(11)—C(10)	106.9 (2)	C(18)—C(17)—H(17)	120.2
O(5)—C(11)—C(12)	106.6 (2)	O(6)—C(19)—H(19A)	109.5
C(10)—C(11)—C(12)	112.5 (2)	O(6)—C(19)—H(19B)	109.4
O(4)—C(12)—C(11)	114.4 (2)	O(6)—C(19)—H(19C)	109.4
O(5)—C(13)—C(14)	119.8 (2)	H(19A)—C(19)—H(19B)	109.5
O(5)—C(13)—C(18)	121.0 (2)	H(19A)—C(19)—H(19C)	109.5
C(14)—C(13)—C(18)	118.8 (2)	H(19B)—C(19)—H(19C)	109.5
O(6)—C(14)—C(13)	116.2 (2)	O(7)—C(20)—H(20A)	109.5
O(6)—C(14)—C(15)	123.5 (2)	O(7)—C(20)—H(20B)	109.5
C(13)—C(14)—C(15)	120.3 (2)	O(7)—C(20)—H(20C)	109.4
C(14)—C(15)—C(16)	119.7 (3)	H(20A)—C(20)—H(20B)	109.5
C(15)—C(16)—C(17)	120.8 (2)	H(20A)—C(20)—H(20C)	109.5
C(16)—C(17)—C(18)	119.8 (2)	H(20B)—C(20)—H(20C)	109.5
O(7)—C(18)—C(13)	115.7 (2)	C(21)—C(22)—H(22)	119.5
O(7)—C(18)—C(17)	123.7 (2)	C(23)—C(22)—H(22)	119.6
C(13)—C(18)—C(17)	120.6 (3)	C(24)—C(25)—H(25)	120.0
C(22)—C(21)—C(26)	118.6 (2)	C(26)—C(25)—H(25)	119.9
C(22)—C(21)—C(30)	120.2 (2)	C(21)—C(26)—H(26)	119.4
C(26)—C(21)—C(30)	121.1 (2)	C(25)—C(26)—H(26)	119.5
C(21)—C(22)—C(23)	120.8 (2)	O(8)—C(27)—H(27A)	109.4
O(8)—C(23)—C(22)	125.2 (2)	O(8)—C(27)—H(27B)	109.6
O(8)—C(23)—C(24)	114.6 (2)	O(8)—C(27)—H(27C)	109.5
C(22)—C(23)—C(24)	120.1 (2)	H(27A)—C(27)—H(27B)	109.5
O(9)—C(24)—C(23)	116.0 (2)	H(27A)—C(27)—H(27C)	109.5
O(9)—C(24)—C(25)	124.8 (2)	H(27B)—C(27)—H(27C)	109.5
C(23)—C(24)—C(25)	119.2 (2)	O(9)—C(28)—H(28A)	110.1
C(24)—C(25)—C(26)	120.1 (2)	O(9)—C(28)—H(28B)	110.0
C(21)—C(26)—C(25)	121.1 (2)	C(29)—C(28)—H(28A)	110.2
O(9)—C(28)—C(29)	107.0 (2)	C(29)—C(28)—H(28B)	110.0
O(10)—C(30)—C(21)	108.3 (2)	H(28A)—C(28)—H(28B)	109.5
O(10)—C(30)—C(31)	109.2 (2)	C(28)—C(29)—H(29A)	109.6
C(21)—C(30)—C(31)	113.6 (2)	C(28)—C(29)—H(29B)	109.4
O(12)—C(31)—C(30)	104.7 (2)	C(28)—C(29)—H(29C)	109.5
O(12)—C(31)—C(32)	107.2 (2)	H(29A)—C(29)—H(29B)	109.5
C(30)—C(31)—C(32)	114.3 (2)	H(29A)—C(29)—H(29C)	109.5
O(11)—C(32)—C(31)	111.5 (2)	H(29B)—C(29)—H(29C)	109.5
O(12)—C(33)—C(34)	118.8 (2)	O(10)—C(30)—H(30)	108.6
O(12)—C(33)—C(38)	121.4 (2)	C(21)—C(30)—H(30)	108.5
C(34)—C(33)—C(38)	119.6 (2)	C(31)—C(30)—H(30)	108.5
O(13)—C(34)—C(33)	115.6 (2)	O(12)—C(31)—H(31)	110.2
O(13)—C(34)—C(35)	124.2 (3)	C(30)—C(31)—H(31)	110.2
C(33)—C(34)—C(35)	120.2 (2)	C(32)—C(31)—H(31)	110.2
C(34)—C(35)—C(36)	119.1 (3)	O(11)—C(32)—H(32A)	109.0
C(35)—C(36)—C(37)	121.7 (3)	O(11)—C(32)—H(32B)	109.0
C(36)—C(37)—C(38)	119.1 (3)	C(31)—C(32)—H(32A)	108.9
O(14)—C(38)—C(33)	114.3 (2)	C(31)—C(32)—H(32B)	108.9
O(14)—C(38)—C(37)	125.4 (3)	H(32A)—C(32)—H(32B)	109.5
C(33)—C(38)—C(37)	120.3 (3)	C(34)—C(35)—H(35)	120.5
C(10)—O(3)—H(3)	109.6	C(36)—C(35)—H(35)	120.4
C(12)—O(4)—H(4)	108.8	C(35)—C(36)—H(36)	119.2
C(30)—O(10)—H(10)	109.5	C(37)—C(36)—H(36)	119.1
C(32)—O(11)—H(11)	109.6	C(36)—C(37)—H(37)	120.4
C(1)—C(2)—H(2)	119.5	C(38)—C(37)—H(37)	120.5
C(3)—C(2)—H(2)	119.4	O(13)—C(39)—H(39A)	109.3
C(4)—C(5)—H(5)	119.8	O(13)—C(39)—H(39B)	109.6
C(6)—C(5)—H(5)	119.8	O(13)—C(39)—H(39C)	109.5
C(1)—C(6)—H(6)	119.5	H(39A)—C(39)—H(39B)	109.5
C(5)—C(6)—H(6)	119.5	H(39A)—C(39)—H(39C)	109.5
O(1)—C(7)—H(7A)	109.4	H(39B)—C(39)—H(39C)	109.5
O(1)—C(7)—H(7B)	109.5	O(14)—C(40)—H(40A)	109.4
O(1)—C(7)—H(7C)	109.5	O(14)—C(40)—H(40B)	109.6
H(7A)—C(7)—H(7B)	109.5	O(14)—C(40)—H(40C)	109.4
H(7A)—C(7)—H(7C)	109.5	H(40A)—C(40)—H(40B)	109.5
H(7B)—C(7)—H(7C)	109.5	H(40A)—C(40)—H(40C)	109.5
O(2)—C(8)—H(8A)	110.0	H(40B)—C(40)—H(40C)	109.5
			
C(7)—O(1)—C(3)—C(2)	−5.8 (4)	O(5)—C(13)—C(14)—C(15)	−174.1 (3)
C(7)—O(1)—C(3)—C(4)	172.3 (2)	O(5)—C(13)—C(18)—O(7)	−3.8 (4)
C(4)—O(2)—C(8)—C(9)	−179.9 (2)	O(5)—C(13)—C(18)—C(17)	174.8 (3)
C(8)—O(2)—C(4)—C(3)	−177.8 (2)	C(14)—C(13)—C(18)—O(7)	−176.3 (3)
C(8)—O(2)—C(4)—C(5)	2.3 (3)	C(14)—C(13)—C(18)—C(17)	2.2 (5)
C(11)—O(5)—C(13)—C(14)	−109.4 (3)	C(18)—C(13)—C(14)—O(6)	177.5 (2)
C(11)—O(5)—C(13)—C(18)	78.1 (3)	C(18)—C(13)—C(14)—C(15)	−1.4 (5)
C(13)—O(5)—C(11)—C(10)	107.7 (2)	O(6)—C(14)—C(15)—C(16)	−179.4 (3)
C(13)—O(5)—C(11)—C(12)	−131.8 (2)	C(13)—C(14)—C(15)—C(16)	−0.5 (5)
C(19)—O(6)—C(14)—C(13)	178.1 (2)	C(14)—C(15)—C(16)—C(17)	1.8 (5)
C(19)—O(6)—C(14)—C(15)	−3.0 (4)	C(15)—C(16)—C(17)—C(18)	−1.0 (5)
C(20)—O(7)—C(18)—C(13)	175.9 (3)	C(16)—C(17)—C(18)—O(7)	177.4 (3)
C(20)—O(7)—C(18)—C(17)	−2.7 (5)	C(16)—C(17)—C(18)—C(13)	−1.1 (5)
C(27)—O(8)—C(23)—C(22)	−7.1 (4)	C(22)—C(21)—C(26)—C(25)	−0.5 (4)
C(27)—O(8)—C(23)—C(24)	171.0 (2)	C(26)—C(21)—C(22)—C(23)	0.1 (3)
C(24)—O(9)—C(28)—C(29)	177.8 (2)	C(22)—C(21)—C(30)—O(10)	150.0 (2)
C(28)—O(9)—C(24)—C(23)	−175.0 (2)	C(22)—C(21)—C(30)—C(31)	−88.5 (3)
C(28)—O(9)—C(24)—C(25)	4.3 (3)	C(30)—C(21)—C(22)—C(23)	−177.6 (2)
C(31)—O(12)—C(33)—C(34)	−118.3 (3)	C(26)—C(21)—C(30)—O(10)	−27.7 (3)
C(31)—O(12)—C(33)—C(38)	66.7 (3)	C(26)—C(21)—C(30)—C(31)	93.8 (3)
C(33)—O(12)—C(31)—C(30)	115.3 (2)	C(30)—C(21)—C(26)—C(25)	177.2 (2)
C(33)—O(12)—C(31)—C(32)	−123.0 (2)	C(21)—C(22)—C(23)—O(8)	178.9 (2)
C(39)—O(13)—C(34)—C(33)	−175.5 (2)	C(21)—C(22)—C(23)—C(24)	0.9 (4)
C(39)—O(13)—C(34)—C(35)	4.6 (4)	O(8)—C(23)—C(24)—O(9)	−0.3 (3)
C(40)—O(14)—C(38)—C(33)	178.1 (3)	O(8)—C(23)—C(24)—C(25)	−179.7 (2)
C(40)—O(14)—C(38)—C(37)	0.8 (5)	C(22)—C(23)—C(24)—O(9)	177.8 (2)
C(2)—C(1)—C(6)—C(5)	0.6 (4)	C(22)—C(23)—C(24)—C(25)	−1.5 (4)
C(6)—C(1)—C(2)—C(3)	−1.4 (4)	O(9)—C(24)—C(25)—C(26)	−178.2 (2)
C(2)—C(1)—C(10)—O(3)	147.5 (2)	C(23)—C(24)—C(25)—C(26)	1.1 (4)
C(2)—C(1)—C(10)—C(11)	−91.4 (3)	C(24)—C(25)—C(26)—C(21)	−0.1 (3)
C(10)—C(1)—C(2)—C(3)	178.2 (2)	O(10)—C(30)—C(31)—O(12)	−60.8 (2)
C(6)—C(1)—C(10)—O(3)	−32.9 (3)	O(10)—C(30)—C(31)—C(32)	−177.7 (2)
C(6)—C(1)—C(10)—C(11)	88.2 (3)	C(21)—C(30)—C(31)—O(12)	178.2 (2)
C(10)—C(1)—C(6)—C(5)	−179.0 (2)	C(21)—C(30)—C(31)—C(32)	61.3 (3)
C(1)—C(2)—C(3)—O(1)	179.5 (2)	O(12)—C(31)—C(32)—O(11)	−57.1 (2)
C(1)—C(2)—C(3)—C(4)	1.5 (4)	C(30)—C(31)—C(32)—O(11)	58.4 (3)
O(1)—C(3)—C(4)—O(2)	1.0 (3)	O(12)—C(33)—C(34)—O(13)	4.8 (4)
O(1)—C(3)—C(4)—C(5)	−179.0 (2)	O(12)—C(33)—C(34)—C(35)	−175.3 (3)
C(2)—C(3)—C(4)—O(2)	179.2 (2)	O(12)—C(33)—C(38)—O(14)	−2.5 (4)
C(2)—C(3)—C(4)—C(5)	−0.9 (4)	O(12)—C(33)—C(38)—C(37)	175.0 (3)
O(2)—C(4)—C(5)—C(6)	−179.9 (2)	C(34)—C(33)—C(38)—O(14)	−177.5 (3)
C(3)—C(4)—C(5)—C(6)	0.1 (3)	C(34)—C(33)—C(38)—C(37)	0.0 (4)
C(4)—C(5)—C(6)—C(1)	0.0 (3)	C(38)—C(33)—C(34)—O(13)	180.0 (2)
O(3)—C(10)—C(11)—O(5)	−64.6 (2)	C(38)—C(33)—C(34)—C(35)	−0.2 (4)
O(3)—C(10)—C(11)—C(12)	178.7 (2)	O(13)—C(34)—C(35)—C(36)	179.7 (3)
C(1)—C(10)—C(11)—O(5)	175.7 (2)	C(33)—C(34)—C(35)—C(36)	−0.1 (4)
C(1)—C(10)—C(11)—C(12)	59.1 (3)	C(34)—C(35)—C(36)—C(37)	0.6 (5)
O(5)—C(11)—C(12)—O(4)	78.0 (3)	C(35)—C(36)—C(37)—C(38)	−0.7 (6)
C(10)—C(11)—C(12)—O(4)	−165.1 (2)	C(36)—C(37)—C(38)—O(14)	177.6 (3)
O(5)—C(13)—C(14)—O(6)	4.8 (4)	C(36)—C(37)—C(38)—C(33)	0.4 (5)

Symmetry codes: (i) *x*, *y*, *z*−1; (ii) *x*−1, *y*, *z*; (iii) −*x*+1, −*y*, −*z*+1; (iv) *x*+1/2, −*y*+1/2, *z*+1/2; (v) *x*−1/2, −*y*+1/2, *z*+1/2; (vi) *x*+1, *y*, *z*; (vii) −*x*+2, −*y*, −*z*+1; (viii) *x*−1/2, −*y*+1/2, *z*−1/2; (ix) *x*, *y*, *z*+1; (x) *x*+1/2, −*y*+1/2, *z*−1/2; (xi) *x*−1, *y*, *z*−1; (xii) −*x*+1, −*y*, −*z*+2; (xiii) *x*−1, *y*, *z*+1; (xiv) *x*+1, *y*, *z*−1; (xv) *x*+1, *y*, *z*+1.

### Hydrogen-bond geometry (Å, °)

**Table:**

*D*—H···*A*	*D*—H	H···*A*	*D*···*A*	*D*—H···*A*
O3—H3···O6	0.819	2.089	2.772 (3)	140.7
O4—H4···O7	0.821	2.461	2.758 (3)	102.5
O10—H10···O13	0.820	2.165	2.878 (3)	145.4
O11—H11···O4	0.820	2.191	2.882 (3)	142.1
O3—H3···O5	0.820	2.44	2.859 (3)	113
O10—H10···O12	0.820	2.32	2.760 (3)	114
O11—H11···O12	0.820	2.36	2.795 (3)	114
.—···..	.	.	.	.
